# Effect of time restricted eating on body weight and fasting glucose in participants with obesity: results of a randomized, controlled, virtual clinical trial

**DOI:** 10.1038/s41387-021-00149-0

**Published:** 2021-01-15

**Authors:** Pamela M. Peeke, Frank L. Greenway, Sonja K. Billes, Dachuan Zhang, Ken Fujioka

**Affiliations:** 1 University of Maryland, Baltimore, MD USA; 2 Pennington Biomedical Research Center, Baton Rouge, LA USA; 3 August Scientific, Encinitas, CA USA; 4 Department of Endocrinology, Scripps Clinic, La Jolla, CA USA

**Keywords:** Obesity, Pre-diabetes

## Abstract

**Background:**

Time restricted eating (TRE) is an emerging dietary intervention for weight loss that is hypothesized to reinforce the metabolic benefits of nightly fasting/ketosis. This pilot study investigated the effectiveness of a daily 14-h metabolic fast (14:10 TRE beginning after dinner, a “fasting snack” at hour 12, and ending with breakfast 14 h later) combined with a commercial weight management program on body weight and fasting blood glucose (FBG) in individuals with obesity. We also investigated the effect of the low-calorie, high-fat, low-carbohydrate, and low-protein “fasting snack” on blood glucose.

**Methods:**

This 8-week, randomized, controlled, clinical trial included men and women (BMI ≥ 30 kg/m^2^) between June and October 2020. Study procedures were conducted remotely. Participants were randomized to 14:10 or 12-h TRE (12:12, active comparator) and prescribed a diet (controlled for calories and macronutrient composition) and exercise program that included weekly customized counseling and support. The primary outcome was change from baseline in body weight in the 14:10 group.

**Results:**

Of the 78 randomized participants, 60 (*n* = 30/group) completed 8 weeks. The LS mean change from baseline in weight in the 14:10 group was −8.5% (95% CI −9.6 to −7.4; *P* < 0.001) and −7.1% (−8.3 to −5.8; *P* < 0.001) in the 12:12 group (between group difference −1.4%; −2.7 to −0.2; *P* < 0.05). There was a statistically significant LS mean change from baseline to week 8 in FBG in the 14:10 group of −7.6 mg/dl (95% CI −15.1 to −0.1; *P* < 0.05) but not in the 12:12 group (−3.1 mg/dl, −10.0 to 3.7; *P* = NS). Both interventions resulted in a larger reduction in FBG in participants with elevated FBG (≥100 mg/dl) at baseline (both *P* < 0.05).

**Conclusions:**

In participants with obesity who completed 8 weeks of the 14:10 TRE schedule combined with a commercial weight loss program, there was statistically significant and clinically meaningful weight loss and improvements in FBG.

## Introduction

Time restricted eating (TRE) is an emerging dietary intervention strategy for weight management^1–4^. Common TRE schedules consist of fasting for 12–18 h each day, beginning in the evening or nighttime^3,5^. Restricting the nonfasting (eating) duration to 12 h or less during waking hours has been shown to improve measures of cardiometabolic health, and is increasing in popularity as a unique method for weight loss^1–4^.

Limiting the portion of the day where eating occurs allows greater opportunity for the beneficial effects of fasting to take place. During the overnight fast, fuel utilization shifts from glucose to ketones, which are produced by the liver from fatty acids^6,7^. Ketogenesis and ketosis are maintained in the absence of dietary carbohydrate^8^. This daily metabolic switch from glucose to ketones reinforces metabolic circadian rhythms, reduces oxidative stress and inflammation, and has numerous other benefits^1–4,8–10^. Studies of TRE suggest that extending the duration of the daily fast to periods longer than 12 h may yield further cardiometabolic benefits^3^; however, daily fasting for durations of 16 h or longer usually necessitates missing a meal, which can reduce adherence^1,2^. Because fasting is difficult by nature, foods designed to mimic the ketogenic effect of fasting (i.e., high fat, low carbohydrate, and low protein) have been demonstrated by Longo and colleagues to have potential in providing relief from the hunger while maintaining ketosis, or metabolic fasting^1,11,12^. A low calorie, high fat, low carbohydrate, low protein, “fasting snack” introduced during the daily fast may be useful in assisting individuals to adhere to longer metabolic fasting durations (i.e., greater than 12 h), during TRE by preventing the metabolic shift from ketones to glucose that occur with consumption of a mixed meal.

TRE in humans and time restricted feeding (TRF) in animals have been shown to produce numerous beneficial effects that include improvements in visceral and total fat mass, glucose tolerance and insulin sensitivity, lipids, blood pressure, appetite, inflammatory markers, and the gut microbiome^2,3,5,13–25^. The cardiometabolic effects of TRE in humans has only recently begun to be explored, and the majority of clinical trials investigating the effects of TRE on body weight are small pilot studies^3^. Most of these clinical trials^5,19,20,22–24,26^, but not all^21,27^, demonstrated that TRE results in weight loss. There are no studies that investigate the effect of engaging in a commercial weight loss program combined with TRE on body weight and cardiometabolic endpoints.

This study investigated the effect of engaging in a commercial weight loss program and a TRE schedule of 14-h of metabolic fasting each day, beginning in the evening immediately after the dinner meal (14:10 schedule), on the change in body weight and FBG in individuals with obesity. A TRE schedule of a daily 12-h fast (12:12 schedule) and same commercial weight loss program was included as an active comparator. We also investigated if eating a high fat, low protein, low carbohydrate “fasting snack” (200 kcal) at hour 12 in the 14:10 group would affect blood glucose levels. The macronutrient composition of the fasting snack was designed to avoid an increase in blood glucose levels and maintain ketosis, or metabolic fasting^1,6,11,12^. The commercial weight loss program included customized meal plans (controlled for daily caloric intake and macronutrient composition), exercise advice, and weekly coaching and troubleshooting sessions.

## Methods

### Ethical considerations

This study was examined and approved by the Argus Independent Review Board (Tuscon, AZ) prior to study initiation. The study was performed in accordance with the principles of the 1964 Declaration of Helsinki, and is registered at ClinicalTrials.gov, number NCT04492930. Written informed consent was obtained from all participants prior to participation in the study.

### Participants

Participants were recruited by phone. Eligible individuals who enrolled in the Jenny Craig® Rapid Results™ program in the United States on or after June 22, 2020 were invited to participate in the study, and were screened for eligibility by the study doctor (PM Peeke). Eligible participants were adult men and women between 18 and 65 years of age with a BMI ≥ 30 kg/m^2^, body weight less than 192.8 kg (425 lbs, the limit of the smart scale), had a tablet/smartphone with a camera and internet access, were not taking any medications for weight loss or diabetes, had no history of serious food allergies, no current eating or severe psychiatric disorders, were not currently taking psychiatric medications, had no special dietary requirements, and were not currently pregnant or breastfeeding. Participants were required to enroll in the Jenny Craig® Rapid Results™ program (enrollment is free) and to purchase 8 weeks of food. Participants received a 50% discount on food as compensation for participating in the study. This study was conducted remotely due to physical distancing regulations and increased the use of telemedicine during the COVID-19 pandemic.

### Study design

This pilot study was a randomized, comparator-controlled, clinical trial comparing a 14:10 TRE (intervention) with a 12:12 TRE (active control) over the course of 8 weeks. Eligible participants were randomized by the study coordinator in a 1:1 ratio by screening day to the 14:10 or the 12:12 groups. An equivalent number of men were assigned to each group. Participants were blinded to the nature of the intervention and control groups. The 14:10 group consisted of a 14-h metabolic fast that began after dinner (between 5 and 8 pm) and ended with consumption of breakfast 14 h later. Participants in the 14:10 group were also instructed to eat a fasting snack consisting of 200 kcal of mixed nuts (18 g fat, 5 g protein, 4 g carbohydrate) 12 h after the start of the fast for 5 days each week. The 12:12 (control) group consisted of a daily 12-h fast that began after dinner (between 5 and 8 pm) and ended with consumption of breakfast 12 h later. No fasting snack was administered in the 12:12 group. Dietary regimens for both the 14:10 and 12:12 groups were based on the Jenny Craig® Rapid Results™ program and were reduced in energy relative to expenditure for baseline body weight (approximately 500–1000 kcal/day deficit). The overall macronutrient composition of the diets was approximately 25–35% fat, 45–55% carbohydrate, and 20–30% protein. Participants were provided with three prepackaged meals and one snack (fruit) per day (JC USA, Carlsbad, CA). Participants were provided with the option for curbside food pick up at the nearest Jenny Craig Weight Loss Center or food could be shipped to their home. Participants were also counseled to engage in physical activity by walking throughout the day and to gradually increase their average daily step count to between 7000 to 10,000 steps each day.

This study was conducted remotely. Study supplies (scale, glucometer, lancets, and glucose strips) were shipped to the participants’ homes and study procedures and assessments were conducted by participants at home. Weekly study visits, including safety and tolerability assessments, were conducted over the phone by the study doctor and trained study and coaching staff. The purpose of these calls was to review the protocol, provide support and guidance, monitor adherence, and monitor for adverse events. Participants were asked about any adverse events or changes to their health or physical function since the previous contact. Sample size calculations were not conducted; results of this study will be used to inform future research.

### Study outcomes

The primary outcome was the change from baseline in body weight for the 14:10 group. The secondary outcome was the change from baseline in FBG for all participants in the 14:10 group. Additional outcomes included the change in body weight and FBG in the 12:12 group, the change in FBG in a subset of participants who had elevated FBG (≥100 mg/dl) at baseline, and the differences in the change from baseline to Week 8 in body weight and FBG between the 14:10 and 12:12 groups. Other study outcomes that were included in the protocol include the change from baseline to Week 4 in body weight and FBG and the change in breath acetone; these will be published elsewhere.

### Study procedures

Participants were provided with an instructional video that detailed all study requirements and provided step-by-step demonstrations for all study procedures. Study coaches were trained by and received as-needed support from the study doctor. For both the 14:10 and 12:12 groups, participants were instructed to begin fasting immediately after dinner at approximately 7:00 pm (acceptable range: between 5:00 pm and no later than 8:00 pm) on Day 0. Additionally, participants in the 14:10 group were instructed to eat the fasting snack 12 h after starting the fast on 5 days each week and to eat breakfast at hour 14. Each week, participants in the 14:10 group were asked if there was any impact of consuming the fasting snack at hour 12 on hunger and satiety. Answer options were: worsened, no change, or some benefit (e.g., decreased hunger, enhanced satiety). Participants were also asked if the fasting snack impacted their ability to successfully wait to eat breakfast until hour 14. All participants were instructed to keep a daily journal noting the time of their first and last meals each day.

Body weight was measured weekly in a fasting state. Participants measured body weight upon awakening and after using the bathroom using a BodyTrace BT005 (BodyTrace, Inc., Palo Alto, CA) cellular-enabled smart scale that automatically uploaded data to the study database. Blood glucose tests were conducted by participants via finger stick with the Abbott FreeStyle Freedom Lite (Abbott Park, IL) blood glucose meter. Participants took pictures of the glucometer screen with their tablet/smartphone, which was then emailed to study staff (the glucometer was programmed to display the date and time of each glucose measurement).

For both groups, blood glucose was measured 2 days per week at 12, 14, and 15 h after starting the fast. In the 12:12 group, participants were instructed to eat breakfast after their 12-h glucose measurement. Participants in the 14:10 group were instructed to have their first meal of the day after the 14-h glucose measurement. For the 14:10 group only, on one day where blood glucose measurements were obtained, they were instructed to eat the fasting snack after their 12-hour FBG measurement. On the other day where blood glucose measurements were obtained, participants did not consume the fasting snack.

### Participant support and compliance

Customized support was provided to participants on an as needed basis via email, text, phone, and video conference calls. Study coaches and the study doctor reviewed participant data on a regular basis and conducted follow-up with participants, where needed for problem solving and to assess compliance. The study doctor was blinded to efficacy measurements during the study. Compliance was assessed by attendance on phone/video calls and providing study measurements (body weight, blood glucose). As part of the Jenny Craig® Rapid Results™ program, participants received weekly one-on-one virtual meetings with a weight loss coach to provide weight loss guidance, motivational coaching, and troubleshooting (for technical issues as well as weight loss support and exercise advice), personalized feedback, custom meal planning, and logistical support.

### Statistical analysis

The changes in body weight and FBG from baseline to Week 8 were analyzed for the population of participants who completed the study (provided a Week 8 body weight and FBG measurement). An exploratory analysis of the change in FBG was conducted in participants who had elevated FBG levels (≥100 mg/dl) at baseline and who completed the study. Sensitivity analyses of the change in body weight and FBG were conducted on the intent to treat (ITT) population, which included all randomized participants, using the last observation carried forward method.

To detect the change from baseline for each group, we used a model-based analytical approach employing an ANOVA model that included the fixed effects of TRE schedules and a covariate effect for gender. Because change variable observations in the 14:10 and 12:12 groups were found to have significantly different residual variances, our models used a generalized Satterthwaite approximation (available in SAS PROC MIXED) in evaluating the test statistics. The estimation of intervention effect at Week 8 employed the restricted maximum likelihood methods. For each target variable, we provided least squares (LS) means for the change at Week 8 and for differences comparing the 14:10 and 12:12 groups. Baseline values and interactions between group and gender were considered but did not contribute significantly to the interpretation of results.

To incorporate changes in body weight and FBG across all 8 weeks of the intervention, we performed a repeated measure analysis on changes at all post-baseline timepoints. The model quality estimator AIC was used in picking unstructured covariance pattern as the optimum in describing the underlying correlation between every two timepoints. The model-based overall average change from baseline for each group and group differences were reported. All analyses were performed with SAS 9.4. (SAS Institute Inc., Cary, NC, USA) with significance level for all the tests set at *P* < 0.05.

## Results

### Participants

**Fig. 1:**
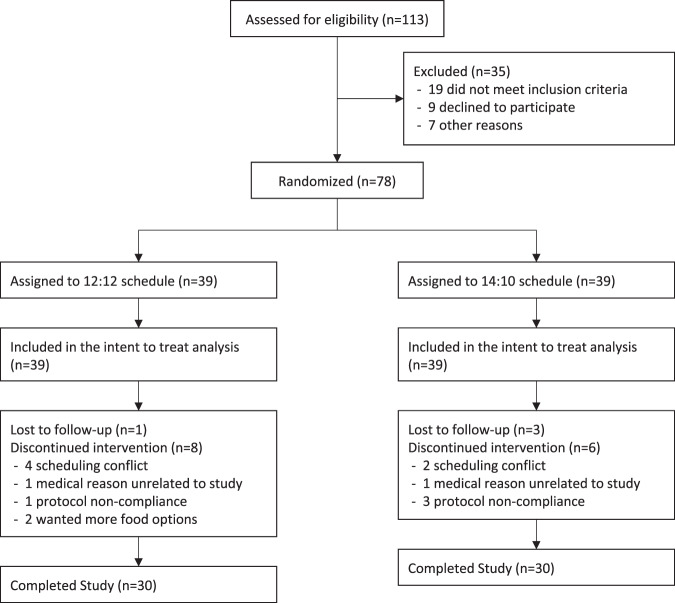
Flow of study participants. Participant flow.

The study took place from June 2020 to October 2020. Seventy-eight participants were randomized and included in the intent to treat population; all provided at least one baseline body weight or FBG measurement (Fig. 1). Sixty participants completed the study (*n* = 30/group) and were included in the analysis of study completers. Reasons for dropouts are summarized in Fig. 1. The medical reasons for leaving the study included a new diagnosis of Parkinson’s disease (12:12 group) and a newly diagnosed food allergy (14:10 group). Six participants dropped out due to a scheduling conflict, i.e., they had to return to work due to changes in COVID restrictions and were no longer able to comply with study procedures.

Demographics and baseline characteristics were generally similar across groups. Most participants were women (88%). Participants who completed the study had a mean ± SD age of 44 ± 11 years and baseline BMI of 38.9 ± 7.7 kg/m^2^. Baseline body weight was slightly higher in the 14:10 group than the 12:12 group but the difference between groups was not statistically significant (*P* < 0.05; Table 1). Baseline data was generally similar between the completer and ITT populations (Tables 1 and 2). Throughout the study, follow-up was provided on a weekly basis and as needed to address patient questions or concerns. There were no substantive differences between groups in the frequency or nature of interactions with the study doctor or coaches between the 14:10 or 12:12 groups.

**Table 1:** Change from baseline in body weight and fasting blood glucose in study completers.

	12:12 Schedule	14:10 Schedule	LS mean difference
Completers population	(*n* = 30)	(*n* = 30)	
Baseline body weight (kg)	121.3 ± 19.1	124.4 ± 20.5	–
95% CI	(114.3, 128.4)	(116.9, 132.0)	
Week 8 body weight (kg)	112.6 ± 18.1	113.8 ± 18.9	–
95% CI	(105.9, 119.3)	(106.8, 120.8)	
Week 8 change in body weight (kg)^a^	−8.9 ± 4.2	−10.7 ± 4.1	1.9 ± 4.5
95% CI	(−10.4, −7.3)	(−12.3, −9.2)	(0.2, 3.5)
*P* value	<0.0001	<0.0001	0.025
Week 8 change in body weight (kg)^b^	−5.4 ± 2.5	−6.8 ± 3.0	1.5 ± 3.6
95% CI	(−6.3, −4.5)	(−7.9, −5.7)	(0.1, 2.8)
*P* value	<0.0001	<0.0001	0.032
Week 8 change in body weight (%)^a^	−7.1 ± 3.4	−8.5 ± 3.0	1.4 ± 3.5
95% CI	(−8.3, −5.8)	(−9.6, −7.4)	(0.2, 2.7)
*P* value	<0.0001	<0.0001	0.027
Week 8 change in body weight (%)^b^	−5.0 ± 1.9	−6.0 ± 1.8	1.0 ± 2.4
95% CI	(−5.7, −4.3)	(−6.7, −5.4)	(0.2, 1.9)
*P* value	<0.0001	<0.0001	0.020
Baseline FBG (mg/dl)	101.5 ± 19.7	102.2 ± 23.6	–
95% CI	(94.3, 108.8)	(93.6, 110.9)	
Week 8 FBG (mg/dl) 95% CI	98.9 ± 14.4 (93.6, 104.2)	95.2 ± 12.9 (90.4, 100.0)	–
Week 8 change in FBG (mg/dl)^a^	−3.1 ± 18.5	−7.6 ± 20.3	4.5 ± 21.3
95% CI	(−10.0, 3.7)	(−15.1, −0.1)	(−3.3, 12.3)
*P* value	0.36	0.047	0.23
Week 8 change in FBG (mg/dl)^b^	−9.6 ± 13.1	−11.0 ± 16.6	1.5 ± 17.6
95% CI	(−14.3, −4.8)	(−17.1, −5.0)	(−5, 7.9)
* P* value	<0.0001	<0.0001	0.65
FBG ≥ 100 mg/dl at baseline (Completers)	*n* = 12	*n* = 12	
Baseline FBG (mg/dl)	113.6 ± 8.7	117.3 ± 21.8	–
95% CI	(108.1, 119.1)	(103.5, 131.0)	
Week 8 FBG (mg/dl)	101.7 ± 12.7	99.1 ± 13.5	–
95% CI	(93.8, 109.6)	(90.7, 107.6)	
Week 8 change (mg/dl)^a^	−11.2 ± 15.8	−17.6 ± 19.3	6.4 ± 22.4
95% CI	(−21.0, −1.4)	(−29.8, −5.5)	(−7.1, 20.0)
* P* value	0.029	0.008	0.33

Data are LS mean ± SD.

*FBG* fasting blood glucose.

^a^ANOVA of the change from baseline to Week 8.

^b^ANOVA of the change from baseline using repeated measures method that included all postbaseline measurements.

**Table 2:** Change from baseline in body weight and fasting blood glucose in the intent to treat population.

	12:12 Schedule	14:10 Schedule	LS mean difference
ITT Population	(*n* = 39)	(*n* = 39)	
Baseline body weight (kg)	121.7 ± 20	125.1 ± 21.2	–
95% CI	(115.3, 128.1)	(118.3, 131.9)	
Week 8 body weight (kg)	113.4 ± 19.9	115.3 ± 20.8	–
95% CI	(107.1, 119.8)	(108.6, 121.9)	
Week 8 change in body weight (kg)	−8.4 ± 5.4	−10.0 ± 5.1	1.6 ± 5.2
95% CI	(−10.1, −6.7)	(−11.6, −8.4)	(−0.1, 3.2)
*P* value	<0.0001	<0.0001	0.063
Week 8 change in body weight (%)	−6.6 ± 4.7	−7.8 ± 4.3	1.2 ± 4.5
95% CI	(−8.1, −5.1)	(−9.2, −6.4)	(−0.2, 2.7)
*P* value	<0.0001	<0.0001	0.094
Baseline FBG (mg/dl)	102.6 ± 32.4	103.8 ± 29.5	–
95% CI	(92.2, 112.9)	(94.3, 113.2)	
Week 8 FBG (mg/dl)	98.1 ± 16.8	96.0 ± 15.4	–
95% CI	(92.7, 103.5)	(91.0, 100.9)	
Week 8 change in FBG (mg/dl)	−3.4 ± 21.2	−8.0 ± 23.3	4.6 ± 23.5
95% CI	(−10.2, 3.5)	(−15.5, −0.5)	(−2.9, 12.1)
* P* value	0.33	0.037	0.22

Data are LS mean ± SD. The intent to treat population includes all randomized participants. Missing data were imputed using the last observation carried forward method.

*FBG* fasting blood glucose.

### Body weight

**Fig. 2:**
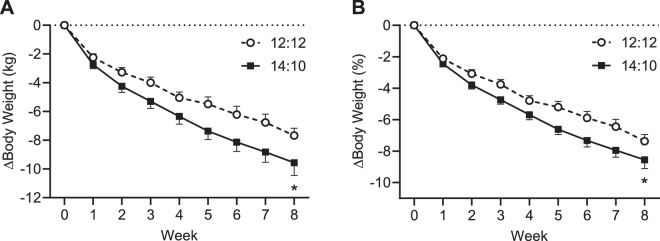
Change in body weight in study completers. **A** Change in body weight. **B** Change in percent body weight. Data are mean ± SE for the completer population (*n* = 30). *P < 0.05 for comparison of the adjusted LS mean change from baseline to Week 8 in the 14:10 group compared to the 12:12 group.

Among participants who completed the study, body weight decreased from baseline to Week 8 in both the 14:10 and 12:12 groups (Fig. 2). Over the course of 8 weeks, the LS mean change from baseline was −10.7 kg (−8.5%) in the 14:10 group (Table 1, *P* < 0.001) and −8.9 kg (7.1%) in the 12:12 group (Table 1, *P* < 0.001). The change in body weight was also significantly different between groups with a between group LS mean difference of 1.9 kg or 1.4% (Table 1, *P* < 0.05). The repeated measures analysis of the change from baseline in body weight yielded similar results (Table 1). The average weight loss was greater than 0 at every timepoint and the overall averages were highly significant for each group. Moreover, the group difference was statistically significant from 0. In the ITT population, the change in body weight from baseline to Week 8 was statistically significant in both the 14:10 and 12:12 groups, but the between groups difference was not statistically significant (Table 2).

### Blood glucose

**Fig. 3:**
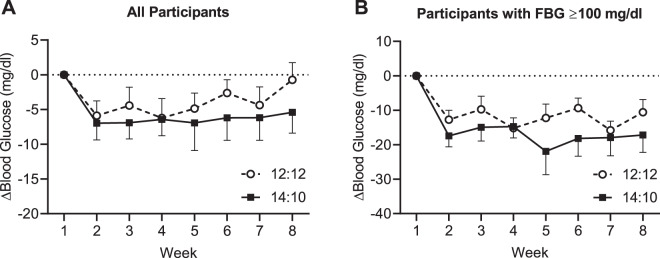
Change in fasting blood glucose in study completers. **A** Change in FBG for all participants. Data are mean ± SE for the completer population (*n* = 30). **B** Change in FBG for participants with baseline levels ≥100 mg/dl. Data are mean ± SE for participants in the completer population with a baseline FBG of ≥100 mg/dl (*n* = 12/group). FBG fasting blood glucose.

Changes in FBG in study completers over the course of the study are shown in Fig. 3A. In the 14:10 group, the LS mean change from baseline in FBG was −7.6 mg/dl from baseline to Week 8 (Table 1, *P* < 0.05). In the 12:12 group, the LS mean change from baseline in FBG was −3.1 mg/dl (not statistically significant, Table 1). The difference in the change from baseline in FBG between the 14:10 and 12:12 groups was not statistically significant (Table 1). The repeated measure analysis showed that the decreases in FBG were significant at all timepoints and for overall post-baseline averages in each group (Table 1). Similarly, the difference between groups was not significantly different from 0. In the ITT population, the change in FBG from baseline to Week 8 was statistically significant in both the 14:10 and 12:12 groups, but the between groups difference was not statistically significant (Table 2).

We analyzed the change from baseline to Week 8 in FBG in subset of participants who had elevated FBG levels (≥100 mg/dl) at the beginning of the study (Fig. 3B, *n* = 12/group). There were statistically significant reductions from baseline in FBG in both groups. The LS mean change in FBG was −17.6 mg/dl for the 14:10 group and −11.2 mg/dl for the 12:12 group (Table 1, both *P* < 0.05).

**Fig. 4:**
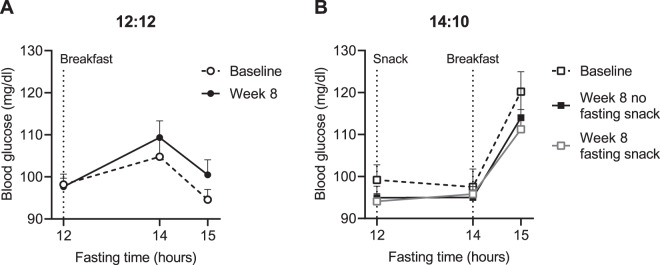
Effect of the fasting snack on blood glucose in study completers. **A** Change in blood glucose after a breakfast meal for 12:12 study completers (*n* = 30). Participants ate breakfast after the 12-hour FBG measurement. **B** Blood glucose response to a fasting snack and breakfast meal for 14:10 study completers at baseline (no fasting snack) and Week 8 (no fasting snack) (*n* = 30). Participants ate the fasting snack after the 12-h blood glucose measurement and ate breakfast after the 14-h blood glucose measurement. Data are mean ± SE.

In the 12:12 group, mean blood glucose levels were similar at hour 12 (immediately prior to breakfast), increased slightly at hour 14 (2 h after breakfast), and decreased back to near fasting levels by hour 15 (Fig. 4A). Week 8 blood glucose measurements were obtained on separate days either with or without the fasting snack (Fig. 4B). Consumption of the fasting snack at hour 12 had no effect on blood glucose levels at hour 14. In the 14:10 group, breakfast was consumed after the 14-hour FBG measurement, which resulted in a significant elevation in blood glucose levels at hour 15 (approximately 1 h after the start of breakfast).

### Effect of the fasting snack

All participants in the 14:10 group reported that the fasting snack decreased hunger and increased a sense of satiety at hour 12. The majority felt that the fasting snack also had a favorable impact on their ability to complete the 14-h fast.

### Safety

There were no reports of adverse events or other safety or tolerability findings.

## Discussion

This virtual clinical trial is the first to examine the effects of combining TRE with a commercial weight loss program that controlled for daily caloric intake and macronutrient composition and provided exercise advice and customized support. By the Week 8 study endpoint, both the 14:10 and 12:12 interventions produced statistically significant and clinically meaningful^28^ weight loss of 11 kg and 9 kg, respectively. Participants in the 14:10 group also exhibited a statistically significant reduction from baseline in FBG of 8 mg/dl at Week 8 compared with a nonsignificant reduction from baseline of 3 mg/dl in the 12:12 group. Individuals with FBG of 100 mg/dl or higher at the beginning of the study exhibited larger reductions in FBG such that the mean blood glucose at Week 8 in the 14:10 group was below 100 mg/dl. For participants who completed 8 weeks, the 14:10 TRE schedule provided a greater reduction in body weight and a numerically greater but not statistically significant reduction in FBG compared with the 12:12 TRE schedule. Similar results were observed in the repeated measures analysis of the completer population. In the analysis of the ITT population, there was no statistically significant superior effect of the 14:10 vs. the 12:12 intervention on body weight or FBG. The ITT population, by definition, includes all participants who were randomized to the intervention (9 out of the 39 randomized participants dropped out of the study in both groups). Thus, the results of the ITT population reflect the efficacy of the intervention in all individuals who attempted the intervention, even if they did not complete the study. Larger studies that are powered to detect between group differences would further address the superiority of the 14:10 compared with the 12:12 schedule.

Although adults in the US report eating over a period of approximately 12 h per day^29^, observational studies have shown that over 50% of adults actually eat during a period of 15 h or longer each day and that they frequently consume the majority of calories later in the day^26,30^. TRE schedules restrict the duration of eating to 12 h or less each 24-h cycle; the most common of which is the 16:8 paradigm. We investigated the effect of a 14:10 schedule because the 16:8 schedule generally requires skipping a meal, which can reduce adherence over the long term^1,2^. Our results indicate that extending the metabolic fast from 12 to 14 h each day may yield greater reductions in body weight and FBG. Furthermore, improvements in FBG were larger in individuals at risk for developing type 2 diabetes (FBG of at least 100 mg/dl). Although earlier studies on TRE have observed mixed effects on glucose and insulin sensitivity^3^, our data are consistent with more recent studies demonstrating that TRE improves glucose tolerance and insulin sensitivity, especially in individuals with greater cardiometabolic risk^3,13,27^. Fat mass, blood pressure, and lipids were not measured in this study because they posed additional logistical hurdles for a virtual study. However, weight loss of at least 5% is associated with clinically meaningful improvements in cardiovascular risk factors such as fat mass, visceral adipose mass, systolic and diastolic blood pressure, LDL-cholesterol, HDL-cholesterol, and triglycerides^1,2^, and have been documented in some studies of TRE^1–5,19,20^.

Preclinical studies demonstrate clear beneficial effects of TRE on glucose tolerance and insulin sensitivity, lipid metabolism, obesity, and the gut microbiome^19^. The benefits of TRE on body weight and cardiometabolic markers in humans have only recently been investigated, and most clinical trials of TRE in adults with obesity are of limited sample size and duration^3,5,15,19,20,22–26^. However, the majority demonstrate that, particularly in individuals with obesity, TRE produces weight loss and enhancements in cardiometabolic health, although the degree of improvements varies across studies and is likely influenced by differences in study population and design.

Preclinical and clinical research demonstrates that many of the beneficial effects of TRE are attributed to reinforcement of metabolic circadian rhythms^1–4,13^. Nutrients play a key role in regulating many body systems such as the autonomic nervous system and endocrine system^20,27^. Thus, erratic eating patterns may disrupt metabolic circadian rhythms^1–3^. It is well established that circadian misalignment (i.e., shift work) is associated with greater risk for obesity, diabetes, and cardiovascular disease^31,32^. The mechanism through which TRE schedules confer protection against obesity and cardiometabolic risk is hypothesized to be due to the metabolic switch from glucose to ketone utilization that occurs with fasting^1–4,6,11,12,32^.

During the overnight fast, fuel utilization shifts from glucose to fatty acids and ketones (ketosis); extending the duration of fasting ketosis contributes to improved glucose regulation^6,7^. We investigated if eating a low calorie, high fat, low protein, low carbohydrate snack at hour 12 of the 14-h fast in the 14:10 group would increase blood glucose levels, which would stop ketosis. The fasting snack contained 200 kcal and was not intended to be a meal replacement. The macronutrient composition of the fasting snack was designed to avoid increasing blood glucose levels and maintain ketosis^6,11,12^. We found that consuming the fasting snack had no effect on blood glucose at hour 14. Furthermore, participants reported that eating the fasting snack favorably impacted sensations of hunger and satiety and may facilitate maintenance of metabolic fasting. Our results are consistent with studies of ketogenic diets, which have been shown to suppress the increase in appetite that is experienced by many individuals during weight loss due to low energy diets^33,34^. Induction of ketosis by a very low energy diet is associated with low concentrations of the hunger hormone, ghrelin, as well as increased levels of the satiety hormones, glucagon-like peptide-1 (GLP-1) and cholecystokinin (CCK)^34^. Thus, the fasting snack may be a useful tool to improve adherence to the 14:10 schedule by reducing hunger/appetite while maintaining the beneficial metabolic effects of fasting.

Participation in a customized weight loss program that includes a controlled diet, increased physical activity, motivational interviewing, and weight loss counseling is known to positively influence weight loss and cardiometabolic outcomes^28,35–38^. The overall weight loss in our study of 7–9% of baseline body weight was much larger than that observed in previous clinical trials of TRE that report modest body weight reductions of 1–3% with TRE compared with ad libitum intake^5,19,20,23,24,27^. Another notable difference of this study was the frequent communication with participants by the study doctor or a trained coach. These regular interactions comprise a substantial part of the commercial weight loss program and likely contributed to the greater effectiveness of the intervention. All participants in both the 14:10 and 12:12 groups received at least weekly communication from a study representative to collect information about study progress, provide logistical, nutritional, and weight loss support, as well as motivational counseling. The substantial weight loss in our study is likely partially attributable to the effectiveness of the weight loss program.

Other aspects of clinical trial design provide explanations for a lack of an effect of other studies of TRE on body weight^3^, as recently demonstrated by Lowe and colleagues^27^ as well as others^21^. The outcomes of both of these studies were likely influenced by a lack of control for daily caloric intake and macronutrient composition^21,27^ and participants may have underestimated daily caloric intake^39^. Lowe and colleagues also reported a decrease in physical activity and a decrease in lean mass in the TRE group^27^. Therefore, reduced protein and higher carbohydrate intake as well as lower physical activity may have contributed to lower lean mass, and further contributed to the lack of an effect of the TRE intervention in this study. Both studies also postponed the first meal by creating a TRE that instructed participants to break the overnight fast at noon^27^ or to delay their first meal by 1.5 h^21^. Increasing evidence indicates that eating later in the day (afternoon/evening) is associated with greater BMI and higher body fat content^30,40^. Furthermore, restricting eating to earlier in the day has a beneficial effect on body weight^26^ and likely accounts for the favorable effect of early TRE schedules^1,2,15,18^, as well as the lack of an effect of later TRE schedules on body weight and cardiometabolic markers^4,15,18^. Thus, TRE schedules such as the 14:10 and 12:12 schedules reported in our study that control for macronutrient intake, encourage physical activity, and reinforce eating earlier in the day will better align with and reinforce metabolic circadian rhythms, thus, producing better cardiometabolic outcomes^4,15,18^.

This study is representative of the current environment in which COVID requirements require physical distancing. All study procedures were performed remotely, resulting in a virtually conducted study. Screening procedures, assistance with equipment set up, troubleshooting, weekly interviews and all follow-ups were conducted over phone/videoconference. Food was made available for curbside pickup or delivered to the participants’ home. Study procedures and electronics were selected to support the conduct of a remote study. Additionally, COVID regulations changed during the conduct of the study and some participants had to return to work during the study, which resulted in some participants dropping out. Thus, changes to the daily routine as well as the difficulty of conducting study procedures (e.g., fasting, eating breakfast, and taking glucose measurements at specific times) contributed to some participant attrition as well as missed measurements.

Some of the study limitations are related to the design of a virtual study, which required study participants to independently conduct study procedures. Prior to study initiation, practice measurements were conducted to familiarize the participants with the equipment and procedures. Week 1 measurements were used for baseline measurements for FBG (and designated as baseline a priori). Because of the remote nature of the study, we did not collect blood (which prevented analysis of lipids and A1C) and did not obtain blood pressure measurements or measure body composition. Thus, the effect of the TRE interventions used in this study on these common cardiovascular risk markers was not assessed. Additional limitations are due to the exploratory nature of the study design. This was a small, relatively short study that was not designed to detect differences between the 14:10 and 12:12 groups. There was a small numerical (and not statistically significant) difference between baseline body weight in the 14:10 and 12:12 groups that may have influenced the study outcome. Additionally, the 8-week study duration was not sufficient to address the long-term effect of the intervention on body weight, blood glucose, and other cardiometabolic markers. As with all lifestyle modifications, it is expected that the durability of the intervention depends on continued adherence, which also remains to be evaluated. Longer and larger studies are needed to verify our findings of between group differences in body weight and blood glucose.

The results of this study demonstrate that statistically significant and clinically meaningful weight loss and FBG reductions can be achieved with TRE combined with a customized weight loss program in as little as 8 weeks. Notably, extending the fasting window from 12 to 14 h each day produced larger reductions in body weight and FBG. Further, larger improvements in FBG were observed in individuals with higher baseline FBG (at least 100 mg/dl). The addition of a low calorie, high fat, low protein, low carbohydrate fasting snack did not affect blood glucose and may be useful in maintaining the fasting metabolic state while improving adherence to the 14:10 schedule. This study supports the utility of TRE paradigms in producing improvements in body weight and metabolic parameters in a weight loss setting.
